# Number selective sensorimotor neurons in the crow translate perceived numerosity into number of actions

**DOI:** 10.1038/s41467-022-34457-5

**Published:** 2022-11-14

**Authors:** Maximilian E. Kirschhock, Andreas Nieder

**Affiliations:** Animal Physiology Unit, Institute of Neurobiology, University of Tübingen, Auf der Morgenstelle 28, 72076 Tübingen, Germany

**Keywords:** Intelligence, Decision

## Abstract

Translating a perceived number into a matching number of self-generated actions is a hallmark of numerical reasoning in humans and animals alike. To explore this sensorimotor transformation, we trained crows to judge numerical values in displays and to flexibly plan and perform a matching number of pecks. We report number selective sensorimotor neurons in the crow telencephalon that signaled the impending number of self-generated actions. Neuronal population activity during the sensorimotor transformation period predicted whether the crows mistakenly planned fewer or more pecks than instructed. During sensorimotor transformation, both a static neuronal code characterized by persistently number-selective neurons and a dynamic code originating from neurons carrying rapidly changing numerical information emerged. The findings indicate there are distinct functions of abstract neuronal codes supporting the sensorimotor number system.

## Abstract (web summary)

Translating a perceived number into a matching number of self-generated actions is key in numerical reasoning. Here, the authors report sensorimotor neurons in the crow telencephalon that signaled the impending number of self-generated actions.

## Introduction

Humans and animals share a primordial and non-symbolic number estimation system^1,2^. It allows them to not only perceive numerosity (i.e., the number of objects in stimuli), but also produce a specific number of self-generated actions^3–5^. The number of self-produced actions contains vital information for wild animals’ decision making. For example, some songbirds produce specific numbers of syllables in their mobbing calls to indicate the dangerousness of predators^6–8^, and the males of certain frog species must match or exceed the number of call syllables of competitors to attract female mating partners^9,10^. Such findings in the wild have been complemented by laboratory studies in which birds and mammals have been trained to perform specific numbers of motor responses^11–14^.

The brain mechanisms representing perceived number have been studied intensively in primates^15–17^. Here, the fronto-parietal network is known as the core number network in which neurons represent sensed numbers^16,18^. The neuronal representation of the number of self-generated actions is much less explored. In monkeys, neurons in the superior lobule of the posterior parietal cortex have been shown to represent different numbers of hand movements in macaques trained to repetitively produce five identical movements^13^. In this study, however, the monkeys had learned to always perform five movements in response to a go-cue; the monkeys were not cued by sensory displays for different numbers of target movements to perform^13^. Thus, the transformation process between sensing a number and producing a number could not be explored. To date, the neuronal processes of transforming perceived number stimuli into a matching number of self-generated actions, the brain mechanisms that link perception to action across time, are unknown^3^. To study this complex process, animals first need to assess numerical information from sensory displays to later perform the perceived target number via self-generated actions.

In the current study, we turned to a corvid songbird, the carrion crow, to investigate the sensorimotor translation in numerical discrimination. Many birds are numerically competent^19–21^, and the associative endbrain area termed *nidopallium caudolaterale* (NCL) is associated with sophisticated avian cognition^22–27^. NCL neurons are selectively responsive to numerosity in visual displays^19,28–30^, even in numerically naïve crows^31^ and newborn domestic chicks^32^, but also encode the planning and execution of goal-directed movements^33,34^. Therefore, the NCL operates at the apex of the perception–action loop in the avian brain and is considered the avian analog of the mammalian prefrontal cortex (PFC)^35–37^. We hypothesized that neurons in the NCL represent the numerical sensorimotor transformation process. In the current article, we report number selective sensorimotor neurons in the corvid NCL as a neuronal correlate of such a numerical perception-to-action transformation mechanism.

## Results

**Fig. 1:**
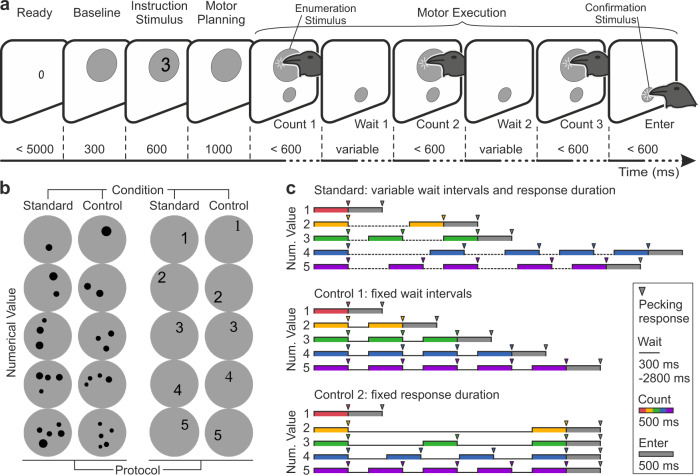
Crows performed an instructed number production task. **a** Number production task. After the crow initiated a trial by positioning its head in front of the touch-sensitive monitor, an instruction stimulus appeared cueing the crow for 1–5 pecking responses (three pecks cued in the example shown). During a brief motor planning period, the crow planned the impending number of pecks. Next, the crow was prompted to peck the instructed number of times, with each unitary peck delivered in a pre-defined epoch cued by enumeration stimuli shown at variable time points. Finally, the crow indicated the end of the response sequence by pecking at a confirmation stimulus (“enter key”). **b** Example instruction stimuli. Each of the numerical values 1–5 was indicated by two stimulus protocols, dot arrays and signs (Arabic numerals). Standard and control stimuli controlled for non-numerical factors in the dot numerosities (position, size, density, and total dot area), and shape appearance (different font types) of the signs, respectively. **c** Temporal arrangement of the response period. To prevent the crows from timing rather than enumerating their pecks, three temporal profiles (standard, control 1, and control 2) with defined response intervals were prompted (see Methods for details). The standard and one of the control arrangements were shown per session, with a pseudo-random trial order.

We trained two carrion crows (*Corvus corone*) to judge the numerical values from one to five in instruction displays and to flexibly plan and perform a matching number of pecks (Fig. 1a). We used displays showing one to five dots (dot protocol) and numerals that the crows had learned to associate with the corresponding numerosity (sign protocol) as instruction stimuli. Both protocols were shown in two stimulus conditions (standard and control conditions; Fig. 1b) to control for non-numerical factors and to promote generalization across instruction stimulus appearance. After the presentation of the instruction numerosity, a motor planning period enabled the crows to prepare the number of pecks. In the following response period, the crows had to generate the number of actions matching the numerical values of the instruction stimulus by pecking on a touch-sensitive screen. To discourage response timing of pecks, each peck was required at pre-defined time points (indicated by enumeration stimuli) interleaved by pauses of variable duration (Fig. 1a). In addition, three different temporal arrangements of the response period were applied to further prevent the crows from using timing strategies to solve the task (Fig. 1c). To indicate the self-chosen end point of the peck sequence, i.e., the final peck amounting to the instructed number, the crows had to peck at a confirmation stimulus that served as an “enter key”.

### Crows produce instructed number of actions

Both crows reliably produced the target number of pecks (Crow 1: 74.7 ± 5.1%, 71 sessions; Crow 2: 72.1 ± 4.0%, 56 sessions; mean ± SD). On average, crow 1 completed more than 400 correct trials per session (mean ± SEM: 410.4 ± 5 hits; *n* = 71 sessions), whereas crow 2 completed more than 300 trials per session (305.4 ± 7.6 hits; *n* = 56). The median pecking reaction times (RT) to the onset of the first enumeration/ confirmation stimuli in the execution period were 444 ms (crow 1) and 389 ms (crow 2), respectively.

**Fig. 2:**
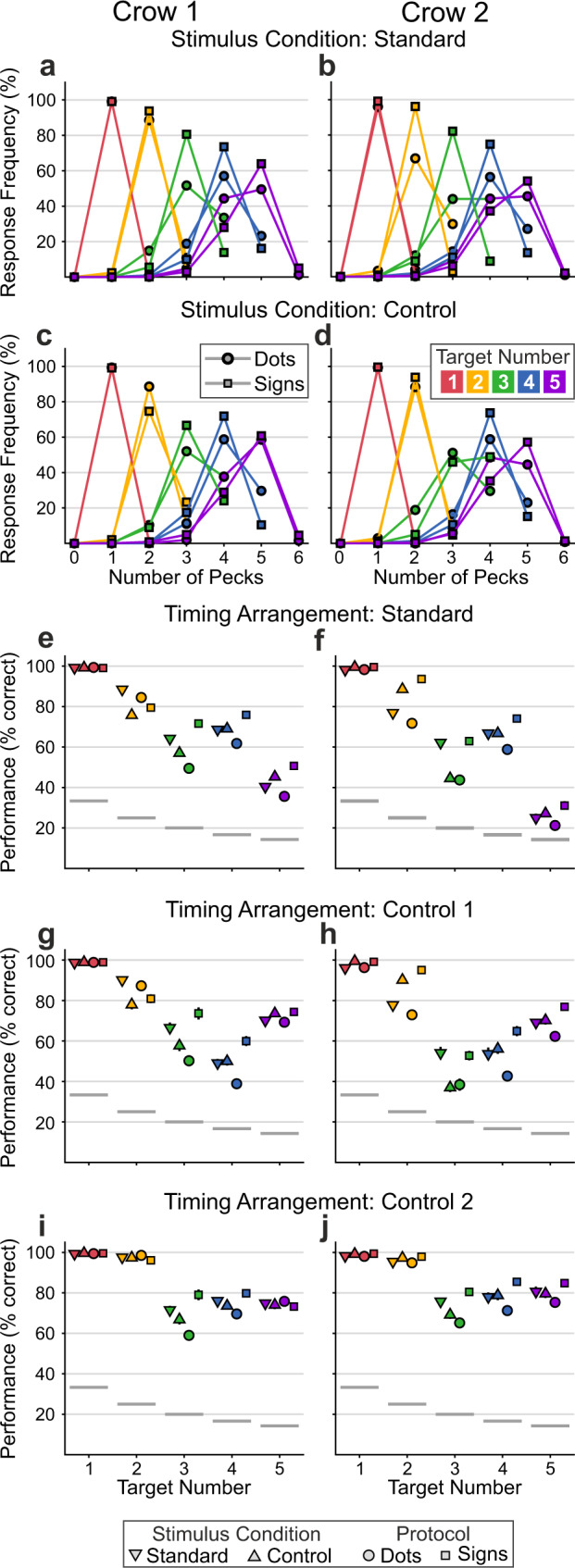
Crows reliably produced the instructed target number of actions. Detailed behavioral performances of crow 1 (**a**, **c**, **e**, **g**, **i**) and crow 2 (**b**, **d**, **f**, **h**, **j**). **a**–**d** Each bell-shaped curve shows the probability that the crows performed a specific number of pecks in response to the instructed numerical value (coded by colors). Peak values represent the respective correct number of responses and off-peak values signify errors. Symbols indicate performance in the dot and sign protocols, respectively. **a**, **b** show performance curves for the standard stimulus conditions, **c**, **d** for the control conditions. Average performances (% correct) of crow 1 (71 sessions) in the standard (**e**), control 1 (**g**), and control 2 (**i**) temporal arrangements of the response period (see Fig. 1c). Performance is shown for all numerical values, stimulus conditions (std. vs. contr.) and protocols (dots vs. signs). Symbol position along the *y*-axis shows the mean, error bars the SEM. Gray horizontal lines in the graphs denote the chance level for each target number (see Methods for calculation of chance levels). **f**, **h**, **j** Average performances of crow 2 (56 sessions) to the different temporal arrangements, numerical values, protocols and conditions (same layout as in **c**, **g**, **i**). Source data are provided as a Source Data file.

The number of correct trials per stimulus protocol and condition combination was overall balanced (mean number of hits for dot/standard, dot/control, sign/standard, sign/control for crow 1, respectively: 94.6, 98.1, 114.2, 103.4; crow 2: 65.6, 72.8, 87.4, 79.6). The performance functions to both stimulus protocols show the peck frequency in response to the instructed numerical value, with peak values typically representing target number and flanking data points representing the frequency of incorrect (too few or too many pecks) responses (Fig. 2a–d). Error rates were highest adjacent to the target number but decreased with distance from it, demonstrating the numerical distance effect^2^. Performance functions became wider (i.e., less precise) with increasing target numbers, a phenomenon known as the numerical size effect^2^. Due to the combined numerical distance and size effects and the resulting worsening of discriminability with increasing number values, nonmatching numbers directly adjacent to larger target numbers (3 and higher) may not have be discriminable significantly by the crows. Both the distance and the size effects characterize an analog number system underlying the crows’ number production behavior^2^.

The performance of both crows to all target numbers, stimulus conditions, and temporal arrangements was above chance (Fig. 2e–j; all *p* < 0.001, one-sided *t*-tests against respective chance levels, *n* = 71 for crow 1, *n* = 56 for crow 2). Note that performance chance levels decrease with increasing target number^38^ (1: 33.3%; 2: 25%; 3: 20%; 4: 16.7%; 5: 14.3%; see Methods for details). Overall, this shows that the crows were able to properly reproduce visually instructed numbers 1 to 5.

### NCL neurons encode the planned number of actions

**Fig. 3:**
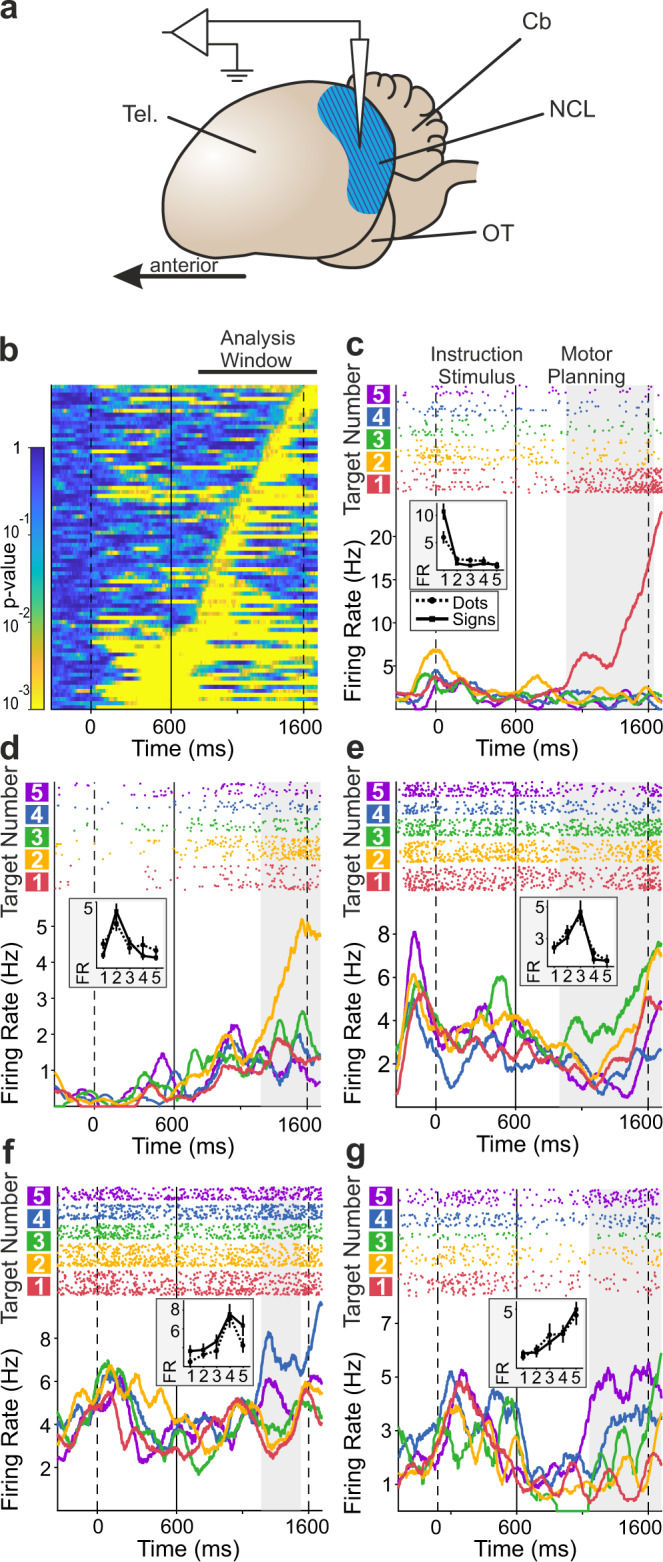
Neurons are selective for the impending number of actions. **a** Lateral view of a schematic crow brain with blue shaded area depicting the recording site *nidopallium caudolaterale* (NCL) in the telencephalon (Tel.). Cb cerebellum, OT optic tectum. **b** Time entries in the motor planning period (600–1600 ms) during which neurons were selective for numerical value (0 ms is instruction stimulus onset). Each line represents the activity of one neuron (*n* = 80), surface color indicates *p* value of selectivity. Thick solid line on top of the surface plot delineates the sliding-window ANOVA analysis interval reported in the main text (800 ms to 1700 ms after instruction stimulus onset to account for neuronal response latencies). **c**–**g** Exemplary neurons selective to numerical values 1–5. Dot raster histograms (each dot representing one action potential) are shown in the top panels with the bottom panels depicting spike-density functions (smoothed with a 150-ms Gaussian kernel). Responses to specific numerical values are color coded. Insets show average tuning curves (to the dot and sign protocols) of the respective neuron during its selective trial interval (indicated by shaded area in the histograms, corresponding to the periods of significant selectivity in **b**). Error bars depict the SEM over trials (*n* = 145, 339, 287, 299, 179 trials overall for neurons in **c**–**g**, respectively). Neurons are tuned to one (**c**), two (**d**), three (**e**), four (**f**), and five (**g**) impending numbers of actions. FR firing rate. Source data are provided as a Source Data file.

While the crows performed this task, we recorded the activity of 339 single neurons in the NCL^39^ (Fig. 3a). We had shown previously in simpler task protocols that many NCL neurons respond to the perceived number of items in visual displays^19,28–30^. Here, we focused analyses on the motor planning period (see Fig. 1a) during which the translation of perceived number into a future number of actions must occur. In this period, many neurons modulated their activity in response to the target number by becoming systematically exited or suppressed to specific numerosities relative to baseline activation.

**Fig. 4:**
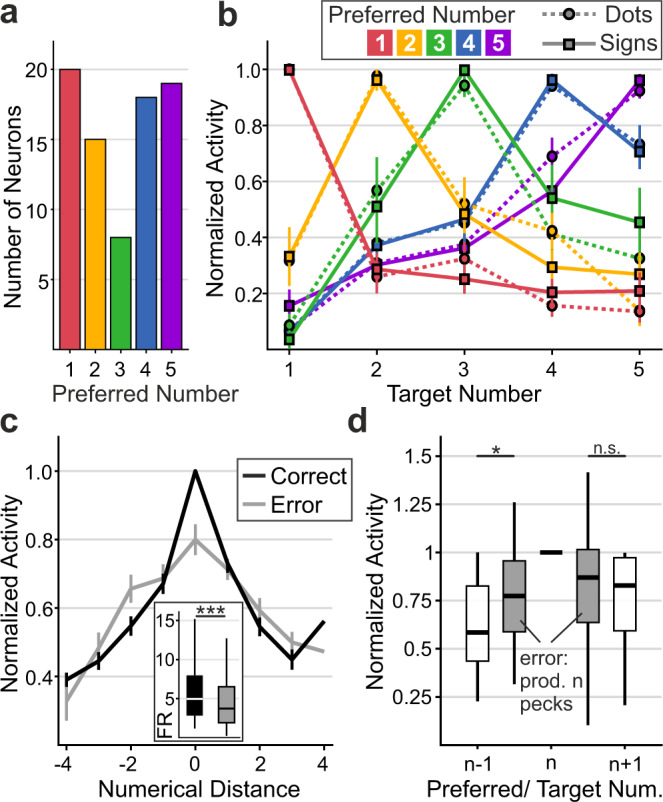
Population of NCL neurons represents the impending number of actions. **a** Incidence of selective neurons preferring each of the one to five impending numbers of actions. **b** Neuronal population tuning functions for the motor planning period. Tuning functions of individual neurons were normalized and averaged according to preferred numerical value (color coded) in both stimulus protocols. Error bars indicate the SEM over neurons (*n* = 20, 15, 8, 18, 19 neurons for preferred numerical values 1–5, respectively). **c** Average neuronal population tuning curves plotted as a function of numerical distance from the respective preferred numerical values (distance 0). The same neurons’ tuning during correct and incorrect trials are shown. Error bars depict the SEM where applicable (*n* = 61 neurons). Inset shows average firing rate to the preferred number during correct and incorrect trials as boxplots (*n* = 61 neurons). **d** Average normalized neuronal activity (*n* = 33 neurons) in trials in which preferred target number *n* was produced erroneously (gray boxes) instead of instructed (*n* − 1 or *n* + 1, respectively) pecking responses (white boxes: activity during correct trials). In Boxplots, the central lines depict the median, bounds of boxes demark the 1st and 3rd quartile, whiskers extend to the minimum and maximum values not considered outliers (>1.5 * interquartile range; not plotted). FR firing rate. Two-sided Wilcoxon sign-rank tests were used. ***p = 10^−8^; *p = 0.02; n.s. not significant (*p* = 0.65). Source data are provided as a Source Data file.

To identify the time intervals in which single neurons selectively responded to the impending number of actions, we employed a well-established statistical procedure^19,28,40–42^. We performed two-factorial sliding-window ANOVAs on the firing rates with factors target number (1–5) and protocol (dots and signs) throughout the motor planning period (criterion *p* < 0.01). This analysis was performed in the time interval from 200 ms after motor planning onset to 1100 ms after motor planning onset to cover the time period after cue instruction and before motor execution (analysis window indicated above Fig. 3b). A quarter of the recorded neurons in both crows (24%; 80/339 neurons) showed a main effect for target number during certain trial times in the motor planning period (Fig. 3b). These number selective sensorimotor neurons showed no main effect for, or interaction with, instruction protocol (dots vs. signs) but were abstractly representing the number of planned motor responses. Number neurons fired maximally to one of the instructed target numbers (a neuron’s preferred number) and decreased firing rates with increasing numerical distances from the preferred number. Individual neurons were tuned to different preferred target numbers (Fig. 3c–g) and covered the full value range from one to five (Fig. 4a). The exemplary neurons depicted in Fig. 3c–g show ‘ramping activity', i.e., they selectively increased firing rates toward the end of the planning period. The normalized and averaged tuning functions of all neurons tuned to a specific preferred target number resulted in overlapping approximate neuronal representations that covered the entire range of target numbers the crows had to plan (Fig. 4b). Neurons responding during the presentation of visual numerosities similarly to the here described numerical planning neurons were found in previous experiments^19,28–31^. The neuronal tuning functions of number neurons tuned in the planning phase mirrored the behavioral performance functions, including the numerical distance and size effects (Fig. 2a–d).

We next explored whether the neuronal activity of tuned neurons was relevant to the correct number of pecks produced by the crows. To that aim, we compared neuronal activity during correct trials and incorrect trials, i.e., trials in which the crows produced fewer or more pecks than instructed. We found that neuronal activity to the respective preferred target number was significantly reduced for incorrect trials compared to activity from correct trials (correct vs. incorrect: 100% vs. 80 ± 4.5%; 6.95 ± 1.03 Hz vs. 5.14 ± 0.62 Hz; *p* < 0.001, *n* = 61 neurons, Wilcoxon signed-rank test) (Fig. 4c). This indicates that the crows were prone to number production errors if number selective sensorimotor neurons were not properly encoding their preferred target number during error trials. Also, the neuronal activity in trials in which the tuned neurons’ preferred number *n* was produced erroneously (instead of instructed number *n* − 1 or *n* + 1) was higher than the activity in trials where *n* + 1 or *n* − 1 was correctly produced (correct(*n* − 1) vs. incorrect(*n*): 61.9 ± 3.8% vs. 72.4 ± 6.1%; *p* < 0.05; correct(*n* + 1) vs. incorrect(*n*): 76.9 ± 3.6% vs. 81.6 ± 5.6%; *p* > 0.5; *n* = 33 neurons, Wilcoxon sign-rank tests) (Fig. 4d).

### Number of actions can be decoded from NCL population activity

**Fig. 5:**
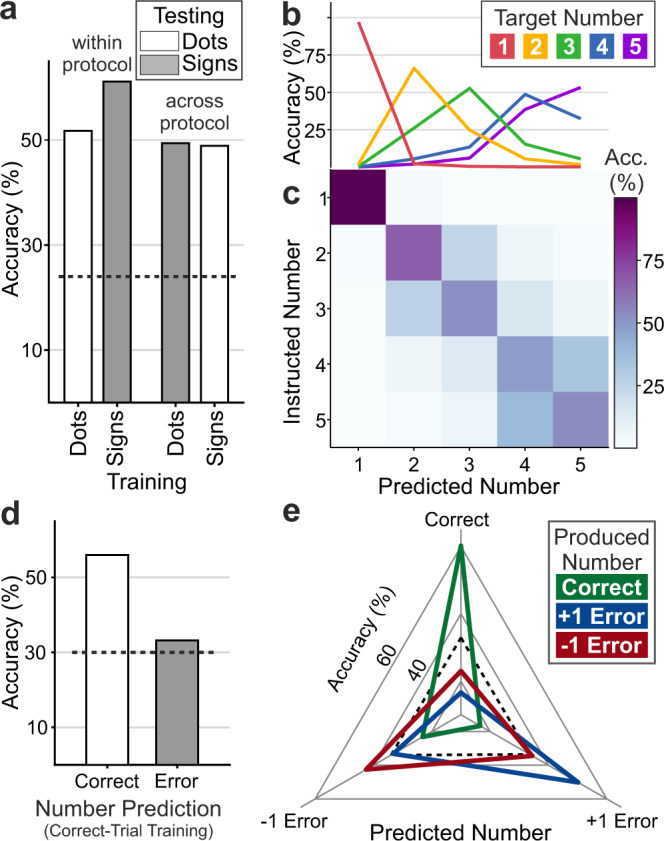
The neuron population predicts the crows’ impending number of pecks. **a** Accuracy of within- and across-protocol classifier prediction performance. Average accuracy for prediction of target number from firing rate of the sampled NCL population during the motor planning period. Within- (trained on trials from a protocol and tested on new trials from the same protocol) and across-protocol performances (trained on trials from a protocol and tested on new trials from the other protocol) are shown. Dotted lines represent 95th percentile of shuffled label classifier performance. Error bars indicating SEM are too small to be depicted. **b** Performance of SVM classifier. Curves show the classification performance for each target number (color coded). Error bars indicating SEM are too small to be displayed. **c** Confusion matrix showing the accuracy of the classifier predicting the number of impending pecks in relation to the instructed number of pecks. The main diagonal describes correct classifications. Values from rows of the confusion matrix result in the curves in **b**. **d** Classifier accuracies for predicting correct and incorrect number of pecks when the classifier was trained on firing rates of correct trials from the motor planning period. Dotted lines represent 95th percentile of shuffled label classifier performance. Error bars indicating SEM are too small to be depicted. **e** Classifier accuracies (spanned in three-dimensional space) for predicting whether the crows would generate less (−1 error) or more pecks (+1 error) than instructed (correct) based on firing rates during the motor planning period. The correct number of pecks and both error types are predicted above chance (33% for three classes; indicated by dotted line). Source data are provided as a Source Data file.

To explore whether the entire population of recorded neurons, irrespective of individual tuning, carried information about the planned number of actions, we trained and tested statistical support vector machine (SVM) classifiers on neuronal activity during the motor planning period (see Methods). First, we trained classifiers on the firing rates for one protocol (i.e., dots or signs) and then used it to classify the planned target number from new trials within the same protocol. We found that within-protocol classification accuracy was well above chance level of 20% for the 5 number classes (dots: 51.7 ± 0.2%, signs: 61.1 ± 0.2%; 270 neurons; mean ± SEM over resamples) (Fig. 5a). We further tested whether a classifier trained on activity of one protocol (e.g., dots) could also predict target numbers for the other protocol (e.g., signs). Indeed, we found robust across-protocol classification (dots to signs: 49.4 ± 0.2%, signs to dots: 48.9 ± 0.2%) (Fig. 5a), indicating abstract and instruction-specific coding of the planned number of actions. With trials pooled across protocols, the neuronal population carried robust information of the target number (63.6 ± 0.2%; 296 neurons; mean ± SEM over resamples). The resulting confusion matrix shows the classifier’s capacity to predict impending target numbers in relation to the instructed target numbers (Fig. 5c); high accuracy values along the diagonal indicated reliable performance. As a reflection of the numerical distance effect, most misclassifications were made in the direct vicinity of the correct number. The tuning curves derived from the classifier performance (Fig. 5b) exhibit a clear distance and size effect mirroring both the crows’ behavioral functions (Fig. 2a–d) and neuronal tuning curves (Fig. 4b). This speaks for a stable sensorimotor population code in NCL that signals the planned number of actions based on the perceived numerical instruction stimuli.

### The population code for prospective number of actions is relevant to behavior

If the sensorimotor population code is relevant for the crows’ behavior, classifier accuracy should predict number production errors and types of errors. Indeed, the accuracy of classifiers trained on correct trials was considerably lower when tested on incorrect trials (33.2 ± 0.3%) as compared to correct trials (56.0 ± 0.2%, 161 neurons; mean ± SEM over resamples; *p* < 0.001, *n* = 1000 resamples, Wilcoxon signed-rank test) (Fig. 5d). Next, we wondered whether the population code could not only predict future correct from incorrect trials, but also the direction of numerical errors, i.e., whether the crows generated fewer (−1 error) or more pecks (+1 error) than the instructed (i.e., correct) number of pecks. We therefore tested classifiers for these three response types across all numerical values (241 neurons; see “Methods” for further details). Indeed, the classifier could predict whether errors were caused by the crows understating (−1 error) or overstating (+1 error) the number of self-generated pecks in relation to the target number (Fig. 5e). Too few pecks were significantly more often assigned to the −1 error class than the +1 error class, and vice versa (*p* < 0.001; Wilcoxon signed-rank tests, *n* = 1000). Thus, already before the motor plan is executed, the population code predicts whether the crows are going to match the target number or miss it by producing too few or too many pecks. Together, these results demonstrate the behavioral significance of the activity of NCL neurons for the sensorimotor translation of perceived into self-generated numbers.

### Dynamic and static codes during sensorimotor transformation

**Fig. 6:**
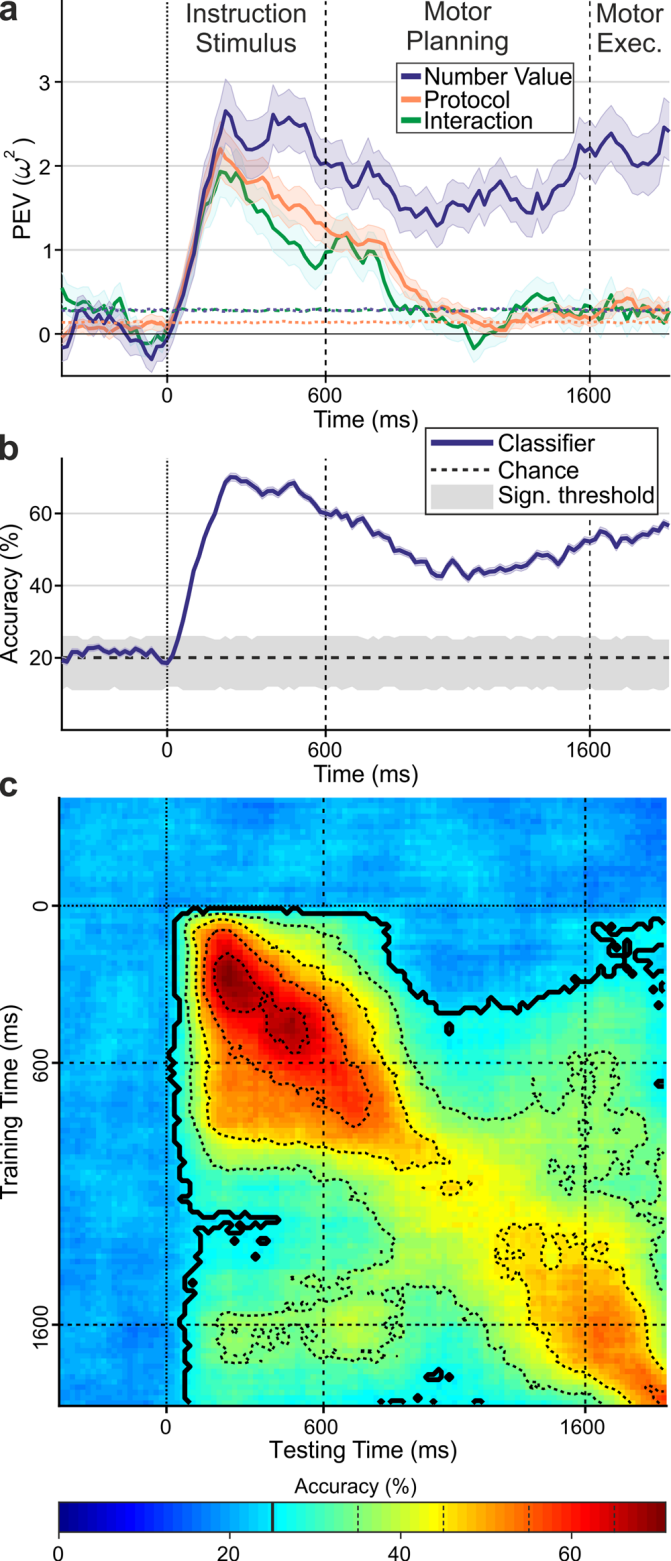
Distinct neuronal codes during sensorimotor transformation. **a** Information (expressed as time-resolved % explained variance; solid lines) about factors target number, instruction stimulus protocol, and their interaction carried by the neuron population across time. Dotted lines show percent explained variance for shuffled trial labels, shaded areas the SEM over resamples. **b** Time-resolved SVM classifier performance. The solid line shows mean accuracy of classifier performance at each point in time during the trial (the mean diagonal accuracy values derived from the cross-temporal confusion matrix shown in **c**, the purple shaded area shows the SEM. The gray shaded area marks the distribution of shuffled label classifier performance (5th to 95th percentile), whereas the dashed line shows the chance level for 5 classes. **c** Cross-temporal SVM classifier performance. Mean accuracy is color coded on the two-dimensional matrix, where training time bins are ordered along the *y*-axis, and testing time bins along the *x*-axis (temporally aligned to **a** and **b**). Straight dotted lines mark the onset of the instruction stimulus period and straight dashed lines indicate the start and the end of the motor planning period. The area outlined by the thick contour line corresponds to the temporal cluster of time bins significantly above chance level (~25%; cluster permutation test, see Methods for details). Dashed contour lines indicate different levels of accuracy (35–65% in steps of 10%). Source data are provided as a Source Data file.

Finally, we explored the temporal dynamics and neuronal code(s) underlying the sensorimotor translation process using two time-resolved neuronal population analyses. First, we performed an *ω²* percent explained variance (PEV) analysis (*n* = 271 neurons; see Methods for details). The PEV quantifies the amount of information about different task factors carried by neuronal populations (Fig. 6a). This analysis showed that neurons initially represented all factors of the instruction stimulus, that is, the instruction target number, but also the stimulus protocol and the interaction between both factors (Fig. 6a). However, sensory instruction information gradually vanished toward the end of the instruction period. After a brief dip in the transition between instruction period and motor planning period, only information about the target number persisted and remained encoded throughout the motor planning period.

We further explored how the neuronal code for numerosity is transformed dynamically into a representation of the upcoming target number of actions by applying cross-temporal classifiers analyses. We trained SVM classifiers on the firing rates from any given time point and tested them during any other time points of new trials. Accuracy was plotted in a confusion matrix spanning the trial times of classifier training against the trial times of classifier testing (Fig. 6c). The accuracy values along the diagonal of the resulting confusion matrix are plotted in Fig. 6b; throughout the entire instruction stimulus period and the motor preparation period, the accuracy values of the classifier were significantly higher than chance. This indicates that the encoded sensory numerosity is conveyed into a planning code for the impending numbers of pecks.

For the sensorimotor transformation process, two extreme codes are conceivable. Neurons might be tuned to a specific target number during the sensory instruction period and maintain this representation over long time periods via persistent firing. This type of coding is known as static coding; here, a decoder trained on neuronal activity during a brief moment of the trial could generalize to other time points of the trial^43^. Alternatively, a dynamic code could occur whereby neurons fire sparsely and rapidly change numerical tuning over time. Here, a decoder trained on neuronal activity during one moment of the trial cannot generalize to the next^43^.

We found evidence for both codes. A static code was evidenced by significant cross-temporal generalization from the beginning of the instruction stimulus period until the beginning of the motor execution period (Fig. 6c).

Thus, classifiers trained on firing rates recorded during the instruction stimulus period were still able to decode the impending number of motor actions when tested on activity recorded during the motor planning period. This resulted in a square-like accuracy pattern in the cross-temporal confusion matrix spanning both trial periods (outlined by thick contour line in Fig. 6c). To test that the cross-temporal generalization and the resulting square-like accuracy pattern was meaningful, we applied a cluster permutation test to the resulting accuracy values (see Methods for details). Indeed, the square-like accuracy pattern (outlined by thick contour lines in Fig. 6c) resulted from accuracy values that were significantly above chance level (~25%; cluster permutation test). This suggests that tuned activity of persistently active numerosity-selective neurons bridged the gap between the sensory instruction stimulus until the beginning of the motor execution period.

We also observed signatures of a dynamic code. Classifiers trained during a specific time interval after instruction stimulus onset showed highest accuracies only within the instruction stimulus period, and—after a performance dip during trial period transition—separately toward the end of the motor planning period. This resulted in highest accuracy values only along the main diagonal of the confusion matrix, with a coding interruption at the onset of the motor planning period (Fig. 6c). The classifiers’ inability to generalize number information across trial time periods indicates an additional dynamic neuronal code in NCL; this is consistent with the finding that many single neurons rapidly changed tuning and encoded target number only during restricted trial intervals (Fig. 3b).

**Fig. 7:**
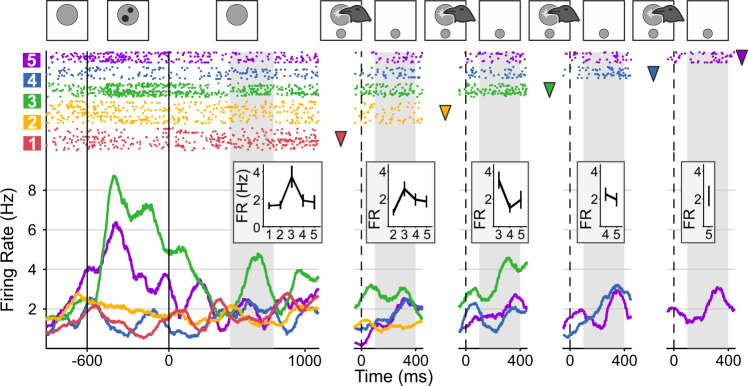
Neuronal activity of an exemplary neuron throughout the entire trial. Exemplary neuron selective to numerical value 3 throughout an entire trial. Dot raster histogram, spike-density functions, and color code are the same as in Fig. 3c–g. Pictograms above the dot raster histograms show the respective task period. Because the time intervals between the crow’s unitary responses in the motor execution period varied (see our timing controls for the different arrangements), the execution responses are temporally interrupted; trials are temporally aligned to previous enumeration pecks (indicated by downward triangle symbols). The gray shaded areas show the time periods over which tuning curves (insets) are summated. Error bars herein depict the SEM over trials (*n* = 111, 111, 68, 61, 51 trials for target number 1–5, respectively). The shaded area during the motor planning period corresponds to the selective interval of this cell (cf. Fig. 3c). FR firing rate. Source data are provided as a Source Data file.

Beyond the instruction stimulus and motor planning period, many NCL neurons were also active during the motor execution period (a detailed analysis of the execution period is beyond the scope of this paper that deals with the sensorimotor translation mechanism). Figure 7 depicts an exemplary neuron that was significantly tuned to number 3 in the instruction, motor planning, and motor execution period. Because the time intervals between the crow’s unitary responses in the motor execution period varied according to the temporal arrangements during the execution period (see Fig. 1c), the execution responses are temporally interrupted. This indicates that the neuronal population actively translated perceived numerical values into numbers of self-generated actions during the motor planning period.

## Discussion

To solve the number production task, the crows used the “approximate number system”, a nonsymbolic number estimation system humans share with a variety of animals^2^. This is evidenced by the approximate bell-shaped performance functions of the crows and the signatures of the psychophysical Weber law, which predicts that their performance functions become systematically broader (i.e., imprecise) with increasing target numbers. Compared to purely perceptual delayed-matching-to-numerosity tasks^19,28–30^, overall performance was decreased in the current delayed number production task. This due to the increasing complexity of the task with increasing target numbers (correlating with decreasing chance performance levels) and the overall increased cognitive demand of the current protocol; the crows not only needed to correctly assess numerosity in the visual displays, but also keep track of their own self-generated actions. Importantly, the performance of both crows to all target numbers, stimulus conditions, and temporal arrangements was above chance. Thus, the crows were able to properly reproduce visually instructed numbers 1–5. For higher target numbers 3–5, performance of both crows was slightly better for the sign protocol (Arabic numerals). This is to be expected as the signs are associated with one precise numerical value, whereas the dot numerosity stimuli provide only approximate numerical values that first need to be estimated perceptually and thus introduce additional noise in number discrimination. Overall, however, the crows’ precision in this task comparable to the one seen in humans asked to reproduce a target number with a series of fast key presses while being prevented from symbolic counting^4,5^.

The crows’ RTs in the execution period indicate that they did not prepare the execution of a peck during the planning period but waited for the onset of the first enumeration stimulus prompting a peck. The crows’ median RTs of 444 ms (crow 1) and 389 ms (crow 2) in the current study were almost identical to the RTs of two crows (394 ms and 460 ms, respectively) that had to peck at the shorter (or longer, depending of the task contingencies) of two choice stimuli, i.e., in a situation where they needed to process the information of the choice stimuli before they could prepare and execute a peck^44^. This indicates that the crows in the current study did not prepare a motor response in the planning period but instead waited until the start of the response phase. The planning period that we analyzed subsequently for representations of impending number of pecks was thus not contaminated by motor-related activity.

The number selective sensorimotor neurons we described in the corvid NCL constitute a neuronal correlate of a numerical perception-to-action transformation process^3^. In the service of this function, NCL neurons fulfilled several expected characteristics: they showed tuning to preferred numerical values, they responded abstractly and irrespective of the format of the instruction stimulus, and most of all, they predicted impending self-generated actions in a behaviorally relevant way. The information (as measured by PEV) contained in the neuronal NCL population robustly encoded the numerical value when the instruction stimulus was shown, but also—mildly diminished—in the absence of a numerical stimulus during the planning period. This information content in the neuron population enabled a classifier to reliably predict the impending number of pecks during the planning period.

The static and dynamic codes we found in the crow NCL during sensorimotor number transformation are not incompatible. In the primate cortex, stable persistent activation with robust across-time generalization is known to exist in the presence of dynamically changing neuronal representations^45,46^. Our results suggest overlaying static and dynamic codes during the transformation of a sensory number code into a representation of a specific motor plan. It is likely that these different codes serve different functions during the transformation process. For example, in the primate cortex, it has been argued that the static code represents retrospective aspects of working memory^47^, whereas a dynamic code might support the prospective nature of working memory to guide future behavior^48,49^. Given that our crows needed to translate an instruction stimulus into numbers of pecks, it is conceivable that the static component resulting from persistently tuned neurons represents the retrospective memorization of the instructed numerical value, whereas the dynamic code stemming from transiently tuned neurons represents the translation of the remembered values into impending numbers of pecks. Interestingly, these two indispensable aspects of the sensorimotor transformation process are represented by a superimposed static and dynamic code, respectively. It stands to reason that overlapping neuronal codes may not only be present in transformations of numerical information, but more generally when sensory input is translated into a volitional goal-directed motor output.

A sensorimotor number system that processes the numerosity of external stimuli but also internally-generated actions was proposed based on psychophysics and brain imaging in humans. Psychophysical findings based on numerosity adaptation after-effects^50^ also suggest a close interaction between numerosity perception and production. For example, participants required to tap rapidly in mid-air for several seconds in an adaptation phase underestimated the number of subsequently displayed visual stimuli near the tapping region; conversely, adaptation to slow tapping caused overestimation of visual numerosity^51,52^. Similarly, motor adaption also changes the perceived numerosity of sequences of auditory tones^53^.

Functional imaging studies in humans have shown that posterior parietal regions activated during the perception of numerical values are found in the vicinity of, and in partial overlap with, action-related (eye or hand movements) areas^54–56^. Indeed, a surprising link exists between the representation of numerical quantities and the preparation of actions^57^. For instance, a decoder trained to distinguish based on fMRI activity leftward from rightward saccades in posterior parietal cortex generalized to distinguish between subtraction and addition^58^. Moreover, one parietal region that contains a topographically organized fMRI-map for numerosities (area NCP3; the third numerosity postcentral sulcus map)^59,60^, seems to be involved in the processing of the number of observed manipulative action^61^. Perhaps the posterior parietal cortex together with other brain regions that harbor numerosity-selective neurons, such as the prefrontal cortex^62–64^ and medial temporal lobe areas^65,66^, constitute the neural substrate of the proposed sensorimotor number system in primates^3^. Similar to the avian NCL, the fronto-parietal neocortical network of primates plays an important role in translating perceptions into actions. Moreover, the fronto-parietal network is known as the core number network in which neurons represent sensed numbers^16^ and number of actions^13^. This cortical network would therefore be ideally suited to enable not only non-symbolic numerical transformations in non-human primates, but also symbolic number transformations during mathematical reasoning in humans.

## Methods

### Animals

Two hand raised male carrion crows (*Corvus corone*), age 2 and 7 years, obtained from the institute’s breeding facility were used in the current experiment. Crows were housed in social groups of up to four individuals in spacious indoor aviaries (L × W × H: 3.6 × 2.4 × 3 m) with daylight and a natural light-dark cycle under controlled temperature and air humidity conditions^67^. Aviaries were equipped with chipped wood bedding on the floor, water trays, wooden perches of varying diameter, and objects for enrichment. A varied diet consisting of birdseed (Beoperlen, Vitakraft, Germany), insect larvae, chick meat, hard-boiled egg, vegetables, fruits and so on was provided.

During the experiments, the crows were kept on a controlled feeding protocol and earned food as a reward during training and recording periods; if necessary, food was supplemented after the daily sessions. Water was provided ad libitum. All procedures were conducted according to the national guidelines for animal experimentation and approved by the national authority, the Regierungspräsidium Tübingen, Germany.

### Experimental apparatus

Training and recording occurred in a darkened operant conditioning chamber. Using leather jesses, the crows were loosely strapped to a wooden perch and placed in front of a 15” touchscreen monitor (3 M Microtouch; 60 Hz refresh rate). A light barrier was used to ensure the crows maintained a central head position in front of the monitor (viewing distance: 14 cm). The barrier consisted of an infrared light emitter/detector fixed on the ceiling of the chamber and a reflector foil attached to the crow’s head. A custom-built automated feeder used for reward delivery was positioned below the monitor. Birdseed pellets (Beo Special, Vitakraft) and mealworms (*Tenebrio melitor* larvae) were used as rewards. Loudspeakers (Visation WB10) for auditory feedback, as well as an infrared camera (Genius iSlim 321R) for observational control, were also installed in the chamber. Presentation of stimuli and collection of behavioral responses was managed by the CORTEX system (Version 595) (National Institute for Mental Health, Bethesda, Maryland). Electrophysiological data was recorded using a PLEXON MAP system (Plexon Inc., Dallas, Texas). Stimulus epochs and behavioral responses were synchronized with electrophysiological recordings with millisecond precision via 8-bit-words as event flags communicated between the CORTEX-system and the Plexon-system.

### Behavioral protocol

We trained two crows on a computerized task to plan and produce a visually instructed number of peck responses. A trial started when a ready cue (small white circle in the center of a touch-sensitive screen) indicated to the crow that a trial could be initiated. To initiate a trial, the crow had to position their head centrally in front of the monitor, thereby closing an infrared light barrier. Moving out of this predefined position before the end of a motor planning period terminated the ongoing trial. Once a trial was initiated, an empty gray background circle was displayed for 300 ms (baseline period). Next, an instruction stimulus (600 ms) instructed the crow on the number of 1 to 5 pecks to produce. This impending number of pecks had to be maintained in working memory throughout the following motor planning period (1000 ms). The appearance of a second smaller gray circle (confirmation stimulus, or “enter key”; size: 11.4° of visual angle) below the empty background circle (now serving as enumeration stimulus; size: 26.1° of visual angle) marked the end of the motor planning period and the beginning of the motor execution period.

In the motor execution period, the crow had to execute the cued number of pecks (one after the other) in a defined way. The crow had to produce each unitary response by pecking within 600 ms at the enumeration stimulus. The enumeration stimulus disappeared after each peck, followed by a short and variable waiting period after which the enumeration stimulus would reappear for as often as the crow would continue to add more pecks (Fig. 1a).

The crow signaled they had made the requested number of responses by pecking at the confirmation stimulus (serving as an “enter key”). Both types of pecking responses (to the enumeration stimulus and the confirmation stimulus) were accompanied by specific sounds (250 ms duration; a high pitched “coin collection” sound from the Super Mario game for a registered enumeration response; a lower pitch “bubble pooping” sound for confirmation responses) serving as auditory feedback for registered responses. A trial was evaluated as *correct* if the number of pecks produced by the crow prior to the confirmation response matched that cued by the instruction stimulus. Correct trials triggered dispensary of a food reward accompanied by a reward tone (an upward FM sweep). If the crow gave a premature confirmation response (produced fewer pecks than the instructed number) or exceeded the requested number of enumeration responses by one (*n* + 1), an error was detected in which the trial was aborted.

If the crow exited the light barrier before the onset of the response period, reacted prematurely during the waiting interval, missed the pecking time interval, or missed the monitor location of the enumeration or confirmation stimuli, the trial was aborted but not counted as error. All errors and trial abortions resulted in the withholding of reward accompanied by a specific sound (250 ms of white noise), a visual feedback signal (a brief, gray full-screen flash), and a brief timeout period (3 s), delaying the initiation of the next trial.

As described below, we used two numerical presentation protocols with a standard and control condition for each numerical value ranging from 1–5. The numerical values, protocols, and conditions were presented in a pseudo-randomized and balanced order.

### Stimuli

We used two different numerical presentation protocols that represented values 1–5 as instruction stimuli: The first protocol (dot protocol) showed a numerosity dot display with 1 to 5 black dots; the second protocol (sign protocol) consisted of five different visual shapes (Arabic numerals) that the crows had learned to associate as signs with the number of actions 1 to 5. Both dot and signs displays were shown on a gray circular background.

For both protocols we used two stimulus conditions (standard and control) to control for non-numerical factors. For the dot protocol, the different stimulus conditions controlled for low-level visual features that covary with numerosity (total dot area and dot density). In the standard condition, dot displays consisted of 1 to 5 dots of pseudo-randomized size (1.2–5.5° of visual angle) presented at pseudo-random locations on the gray background circle, with the only requirement that dots were not overlapping or touching. In the control condition, total dot area and density was kept constant across numerical values.

For the sign protocol, black Arabic numerals 1–5 of pseudo-random size (15–26 pts., 2.9–4.9° of visual angle) were placed at a pseudo-random location on the background. “Arial” was used as the standard font-type, whereas “Times New Roman”, “Souvenir”, and “Lithograph Light” were used as control fonts. To prevent the animal from memorizing or rote learning individual stimuli, multiple stimuli for every combination of protocol, condition, and numerosity were generated anew before each session using MATLAB (Version R2020b, MathWorks Inc., Natick, Massachusetts).

### Temporal arrangement during the enumeration period

The duration of the wait intervals in the motor execution period was varied systematically to prevent the crows from using timing strategies to solve the task. We applied three temporal arrangements: one standard and two control arrangements (Fig. 1c). In the standard timing arrangement, the duration of each wait interval was chosen pseudo-randomly between 300 ms and 1.2 s (in steps of 300 ms). Each of the interval durations had an equal probability per wait interval, therefore, rhythmicity was suspended. Although response period duration increased with requested numerosity, it overlapped between neighboring numerosities (see Fig. 1c). In the first control timing arrangement (fixed wait interval), all wait intervals had a fixed duration of 300 ms. In the second control protocol (fixed overall duration), wait interval durations varied according to the requested numerosity, such that the total duration of the response period was the same across numerosities. Therefore, wait interval durations were shorter for higher numerosities and vice versa, i.e., trials with requested numerosity two had one 2.8 s wait interval, numerosity three had two 1.2 s intervals, numerosity four had three 600 ms intervals, and numerosity five had four 300 ms intervals. Since trials with numerosity one had no wait interval, these trials had the same temporal organization.

For each session, the standard timing arrangement and one of the two control timing arrangements were presented in pseudo-random and balanced order; the control timing arrangements were alternated daily.

### Training procedure and learning criterion

Prior to training on this task, crow 1 was experienced with numerosity discrimination in a delayed match-to-sample protocol, whereas crow 2 was naïve. The layout of the current task was introduced in a step-wise manner by progressing from one training step to the next over the course of months; once the crows were proficient with one training step, they were moved to the next step.

In a first step, the crows were shown an instruction stimulus containing two black dots. After a brief delay, two sequential white squares appeared in the motor execution period. The crows had to peck at each white square (which disappeared after being pecked) to receive a reward. In a second step, an instruction stimulus containing one dot was introduced. In addition, the “confirmation stimulus” was introduced during the motor execution period. Moreover, an “enter cue” (the same white square that served as count cue) was turned on within the confirmation circle once the required number of pecks to the enumeration stimulus was registered. Now the crows received a reward when it pecked at one or two white squares (depending on the cued number) and then on the enter cue. Target numbers one and two were first presented in blocks of trials and later interleaved once the crows mastered them individually. In a third training step, the white squares were faded out in color against the gray background until they were invisible, leaving the gray background circle as “enumeration stimulus”. At this stage, short wait intervals without an enumeration stimulus were introduced between enumeration pecks to ensure the crows emitted discrete pecks. By the end of this step, the crow was able to produce one or two pecks and to confirm using the confirmation stimulus as enter key. In the fourth training step, the display time of this enter cue was then shortened until it was only briefly flashed (after the crows produced the correct number of pecks) and finally not displayed at all. The crows were now pecking at the confirmation stimulus upon correct number of, one or two, pecking responses unaided by any cues other than the instruction stimulus. In the following training steps, target numbers 3–5 were introduced one by one in the same block-wise manner as described above. For example, the crow was presented with only target number 3 trials until it—by chance—produced three pecks to the enumeration stimuli and got rewarded upon pecking the enter key subsequently. Once the crow proficiently produced target numbers 1–5 for the standard dot protocol, the control condition stimuli for dots were introduced.

After that, the sign protocol was introduced. This was done by initially showing two instructing stimulus phases, first the dot stimulus followed by the corresponding sign stimulus. The crows thereby learned to associate dot numerosities with the respective sign stimulus until the dot numerosities could be removed to only leave the sign as instruction stimuli. After the crows mastered the sign protocol with target numbers 1–5, control condition sign stimuli were introduced. Finally, trials showing dot and sign instruction stimuli were gradually interleaved. The last training step consisted of introducing the different temporal arrangements, until the crows proficiently mastered the standard and the control 1 and control 2 temporal arrangements in the motor execution period.

The crows were trained on the final protocols until they reached the learning criterion. The learning criterion was reached when the crows performed all five numerical target values and all three temporal arrangements above chance for 9 consecutive sessions (i.e., days). Chance levels decreased with increasing target numbers: 33.3% for number 1 (given that the crows could perform 0, 1, or 2 pecks before using the confirmation stimulus as “enter key”, i.e., 3/100%); 25% for number 2 (either 0, 1, 2, or 3 pecks; 4/100 %), 20% for number 3, 16.7% for number 4, and 14.3% for number 5.

### Surgery and recordings

After crows reached the learning criterion, we implanted custom-made micro-drives carrying electrodes for electrophysiological recordings. All surgeries were performed while the animals were under general anesthesia. Crows were anesthetized with a ketamine/xylazine mixture as described in Ditz and Nieder^19^. The head was placed in the stereotaxic holder that was customized for crows with the anterior fixation point (i.e., beak bar position) 45° below the horizontal axis of the instrument. Using stereotaxic coordinates (center of craniotomy: anterior-posterior +5 mm relative to inter-aural (ear bars) as zero; medial-lateral 13 mm relative to midline), we chronically implanted two micro-drives with four electrodes (spaced apart ~0.5 mm) each in the left or right hemispheres, a connector for the head stage and a small head post to hold the reflector for the light barrier. Glass-coated tungsten microelectrodes with 2 MΩ impedance (Alpha Omega) were used. The electrodes targeted the corvid NCL, which is characterized by dopaminergic cells^39,68,69^. After the surgery, the crows received analgesics^19^.

When the crows had fully recovered a few days after the surgery, single-unit recordings in the behaving crows commenced. We recorded from both hemispheres of both crows (9 sessions from right NCL and 62 sessions from left NCL in crow 1; 5 sessions from left NCL and 51 sessions from right NCL for crow 2). Each recording session started with advancing the electrodes until a proper neuronal signal (of at least 3:1 signal to noise) was detected (see also Fig. 4a, b in Veit and Nieder^68^, for example recording traces). Neurons were not preselected in the involvement of the task.

Every session the birds were placed in the recording setup and a head stage containing an amplifier was plugged into the connector implanted on the bird’s head and connected to a second amplifier/filter and the Plexon MAP box outside of the setup by a cable above and behind the bird’s head (all components by Plexon Inc., Texas, USA). Signal amplification, filtering, digitizing of spike waveforms was performed using the Plexon system. Spectral filtering of recordings was accomplished by a combined preamplifier filter (150 Hz-8kHz, 1 pole low-cut, 3 pole high-cut) and main filter (250 Hz, 2-pole, low-cut filter). Amplitude amplifications were set individually for different channels in the range of ca. 20,000x gain. Spike waveforms were sampled at a frequency of 40 kHz. Plexon’s Offline Sorter was used to manually offline sort spikes into single-unit waveforms by applying mainly principal component analysis.

We verified the NCL and the location of the electrodes in NCL (according to the implantation coordinates provided above; as histologically verified before, see ref. 70). We immunohistochemically stained for tyrosine-hydroxylase to identify dopaminergic cells, which characterize the NCL^71^ (see also Fig. 3 in Veit and Nieder^68^).

### Data analysis

All analyses were carried out in MATLAB (Release 2020b), unless stated otherwise. If not stated otherwise, all values in the figures and main text refer to the mean ± standard error of the mean (SEM). The latter was calculated as the standard deviation divided by the square root of sample size.

### Behavioral analysis

Overall performance (% correct) was calculated as the number of correct trials divided by the sum of correct and incorrect trials for each session. To construct performance curves, the relative frequency of number of responses made to the enumeration stimuli were calculated for each instruction numerical value (separately for dot and sign protocols) and averaged over recording sessions.

### Neuronal tuning and selectivity analysis

Single units were included in the analyses if they had an average firing rate of at least 0.5 Hz in the relevant task windows (beginning of baseline period until end of motor planning period), and at least two recorded correct trials for each of 20 specific trial conditions (5 numerical values × 2 stimulus protocols × 2 stimulus conditions).

Neuronal activity in the motor planning period was evaluated in a 900 ms time window from 800 ms to 1700 ms after instruction stimulus onset (200 ms to 1100 ms after onset of the motor planning period). With this, the analysis window reached 100 ms into the physical presentation of the first enumeration response period but based on the neurons’ response latency allowed us to still acquire activity related to the crows’ planning. The mean visual latency for crow NCL neurons is 144 ms^70^. Therefore, the neuronal activity relative to the physical task periods is delayed by this amount of time (i.e., 144 ms, on average). Thus, the first 100 ms of the execution period still carry only information about the planning period, not the motor execution.

We used two-factor sliding-window analyses of variance (ANOVA; 200 ms window, 10 ms step-size, *p* < 0.01) in this 900 ms window to assess single-neuron selectivity to the task variables “numerosity” (numerical values 1–5) and “notation” (dots and signs). As stimulus condition (standard and control) differed for dot and sign protocols, it was not included as a factor into the ANOVAs. If neuronal activity differed significantly for numerical value (i.e., if there was a significant main effect for factor numerical value, but no main effect for protocol, nor a significant interaction term), in more than 11 consecutive time-bins (amounting to a continuous interval of at least 300 ms), this interval and the neuron were categorized number-selective. In case there was more than one selective interval for a neuron, only the one containing the largest difference in firing rate for different numerical values was used.

For every number-selective neuron and its selective interval, the numerical value eliciting the highest firing rate was termed its preferred numerical value. Neuronal response functions were constructed by normalizing a neuron’s mean discharge rate between it most- and least-preferred numerical value. Population response functions were obtained by averaging tuning function of all number-selective neurons preferring each individual numerical value.

### Error trial analysis

To infer behavioral relevance of the motor planning activity, the discharge rates in response to instructed numerical values were compared between correct and incorrect trials. Only number-selective neurons with at least three recorded incorrect trials for each instructed numerical values were considered in this analysis. The individual neuron’s mean firing rates to their preferred numbers were compared in correct vs. incorrect trials (Wilcoxon signed-rank test). Furthermore, neuron’s response functions were calculated for correct and incorrect trials and were overlayed by aligning them to their preferred numerical values. To compare the tuned neurons’ responses in trials where the preferred number *n* was produced erroneously, we considered only a subset of number-selective neurons. Neurons had to have at least three recorded trials in which *n* pecks were incorrectly produced instead of the *n* − 1 and *n* + 1 instructed pecks. Consequently, only neurons tuned to preferred target numbers 2, 3, and 4 were considered in this analysis. Additional error analyses were performed at the population level (see below).

### Classifier analyses

We performed classification analyses for cued target numerical values using linear multi-class support vector machine (SVM) classifier models^72^ trained and tested on trial firing rates within the 900 ms motor planning period analysis window. To evaluate auto-classification accuracy for the entire sampled population, we only considered neurons with at least 20 correct trials per stimulus class, i.e., numerical value. To deal with the problem of multiclass classification arising from our five classes, we used one-vs.-one transformation to binary classification provided by the used models^72^. In brief, each multiclass classification was split into 10 binary classifications (i.e., class 1 vs. class 2, 1 vs. 3, 1 vs. 4, and so on), onto which the model was trained to discriminate. This one-vs.-one approach is commonly preferred over the alternative one-vs.-rest approach (class 1 vs. classes 2–5, 2 vs. 1 and 3–5, etc.). To evaluate auto-classification accuracy (for which training and test data stem from the same dataset), we performed a 10-fold cross-validation approach: we divided the data (average normalized firing rates for each of the 5 classes) into 10 even splits. We then used 9 of these splits to train a classifier model, and used the 10th split for testing the model. In particular, the normalized average firing rates of 90 trials (18 trials per 5 classes) were used to train an SVM model, and the models’ predictions of the remaining 10 trials (2 trials per 5 classes) were then evaluated to yield an accuracy measure of this cross-validation run. This was repeated 10 times overall, each time with another of the 10 splits as the testing subset. Prior to training and testing, trial firing rates were *z*-scored, that is, subtracted with the mean and divided by the standard deviation of the training data (these parameters of the training subset were also used to *z*-score the testing subset of trials). We repeated this procedure 1000 times, each time with a new subset of 20 randomly drawn trials for each neuron. To assess chance level classifier performance, we also repeated the above procedure (including *z*-scoring, cross-validation splits, and resampling) with shuffled trial labels. The classifier model predicts the class label of a given subset of trials. Together with the correct labels, we used predicted labels to construct confusion matrices. Overall classifier accuracy was calculated as the average over the mean diagonal of the confusion matrix. Values in the figure and main text report the mean and standard error of the mean (SEM) over resamples.

We used an analogous approach to test whether the neuronal population would transfer information about the target number across different stimulus protocols (dots or signs). To this end, we trained SVM models on the classification of numerical value in one protocol and tested in on the other protocol. This process is very similar to the aforementioned analyses; hence, we only mention deviations from the above procedure. We included all units that were recorded for at least 10 trials per numerical value and stimulus protocol (10 trials for each numerical value in the dot protocol and 10 for each numerical value in the sign protocol). We tested how well SVM models trained on one stimulus protocol (e.g., dots) could predict the numerical value of trials of the other protocol (e.g., signs; and vice versa). Similar to a 10-fold cross-validation, nine trials per class of one protocol were used for training and one trial per class of the respective other protocol were then used for testing the SVM models per split. This was repeated 10 times, each time with another split of trials for training and testing. All other parameters of this approach (i.e., normalization, resampling, chance level performance) were identical to the auto-classification described above.

This classification/prediction approach was also used to demonstrate behavioral relevance of the population signal by testing it on activity from incorrect trials. Analogous to the above, we included all neurons that had at least one incorrect trial for each class. This number was small because there were very few incorrect trials for the numerical value 1. Nine correct trials per class were used for training and either one correct or one incorrect trial per class were then used for testing the SVM models per split. Again, this was repeated 10 times, each time with another split of trials. All other procedures for this classifier were identical to the analyses above. We thus yield two accuracy measures from this analysis: one for the auto-classification of correct trials, and one for the prediction of incorrect trial labels from models trained on firing rates of correct trials.

Lastly, we also checked whether the population carried information about the trial outcome. We trained and tested an SVM classifier on whether a trial would be correct, incorrect due to prematurely ending the enumeration period (−1 error), or incorrect due to overshooting the target number of enumeration pecks (+1 error). We considered only −1 errors (instead of for example −2 errors) to balance out the fact that only +1 errors were possible due to the task design. That is, an ongoing trial would automatically be aborted after *n* + 1 pecking responses (for target number *n*). We included all recorded neurons that had at least 30 trials of these three classes irrespective of the numerical value. This was done because there were vastly different numbers of +1 and −1 errors for different target numbers. For example, there were virtually no −1 errors for target number 1. We used a 10-fold cross-validation, and resampled trials 100 times. All other steps were identical to the auto-classification of numerical value described above.

### Time-resolved population analyses

To determine the amount of information about numerical values and stimulus protocol carried by the neurons over the time course of a trial, we performed a percent explained variance (PEV) analysis^73,74^. We^29,62,75^ and others^76,77^ have used the PEV analysis before to extract the amount of information carried by neuronal populations. The PEV captures the amount of information represented in the firing rates of the population irrespective of selectivity. It reflects the amount of variance in the firing rates explained by the task factors (“target number” or “stimulus protocol”). To this end, all recorded neurons with at least 10 trials per numerical value and stimulus protocol were considered. We used two-factorial (numerical value, stimulus protocol) sliding-window ANOVAs (200 ms window with 20 ms step size) over the relevant time window of the trial (baseline onset until motor planning offset; 300 ms prior to instruction stimulus onset until 1700 ms after instruction stimulus onset) to yield the sum-of-squares. From these, the amount of variability attributed to either factor or their interaction as a function of time was calculated as *ω*² according to:

1 $${{{{{{\rm{\omega }}}}}}}^{2}=\frac{{{{{{{{\rm{SS}}}}}}}}_{{{{{{\rm{term}}}}}}}-\,{{{{{{\rm{df}}}}}}}\times \,{{{{{{{\rm{MS}}}}}}}}_{{{{{{\rm{error}}}}}}}}{{{{{{{{\rm{SS}}}}}}}}_{{{{{{\rm{total}}}}}}}+\,{{{{{{{\rm{MS}}}}}}}}_{{{{{{\rm{error}}}}}}}}\times 100$$

where SS_term_ is the sum-of-squares of the term of interest (factors number or protocol, or their interaction), SS_total_ the total sum-of-squares, df the degrees of freedom, and MS_error_ the mean squared error. Values were averaged over units to extract the population PEV value as a function of time for each term. We repeated this procedure 20 times, each time with randomly drawn trials. Average PEV and SEM values are based on the mean over resamples. To assess baseline PEV, we also performed the procedure with shuffled trial labels in the ANOVAs, shuffled 50 times per resample (20 resamples × 50 shuffles = 1000 reshuffles for the calculation of baseline PEV).

To further quantify the sensory-to-motor transformation of the population code, we also performed a cross-temporal SVM classifier analysis. For this, we considered only neurons that were recorded for at least 20 correct trials per class, i.e., target number. We used a sliding-window (200 ms length, 20 ms step size, from baseline onset to the beginning of the motor execution period) and trained a linear multi-class SVM model in each of these windows. Similar to a 10-fold cross-validation, we split the data in 10 equal parts, that is, we used firing rates of 18 trials per class in the respective time window to train the model and then tested each window’s model with firing rates of the remaining two trials per class from each of the remaining time windows. This way, a two-dimensional matrix of accuracy values—where the first dimension describes the time bin for the training of the classifier model, and the second dimension the time bin for testing—is yielded. These training and testing procedures were repeated 10 times, each time with a different split of trials. One-vs-one classification was used to deal with five classes. Before training and testing, firing rates were z-scored based on parameters from the training subset. The whole procedure was repeated 20 times, each time with a different subset of randomly sampled trials.

To assess chance level accuracy of the cross-temporal classifier, we performed a cluster permutation test^78^. We repeated the procedure (including 10-fold cross-validation and *z*-scoring) with permuted trial labels, shuffling 50 times per resample (50 shuffles × 20 resamples = 1000 reshuffles for chance level accuracy). We then compared the mean (over resamples) of true accuracy values against the distribution of random values in each time bin (pixel in the 2D accuracy matrix; *α*_cluster_ = 5%). This was also repeated for each of the (1000) permuted accuracy matrices. In a second step, neighboring pixels significant above this first threshold formed so-called “candidate clusters”. Cluster size, i.e., the number of neighboring pixels of these candidate clusters (of both, true data, and shuffled data) were then used to form a distribution, against which the true accuracy clusters were evaluated for significance (*α*_rank_ = 5%).

### Reporting summary

Further information on research design is available in the Nature Portfolio Reporting Summary linked to this article.

## Supplementary information

Reporting Summary

## Data Availability

The data that support the findings of this study are available from the corresponding author upon request. Source data are provided with this paper.

## Source data

Source Data

## Code availability

The code that support the findings of this study is available from the corresponding author upon request.
