# Supplementary material for: Number selective sensorimotor neurons in the crow translate perceived numerosity into number of actions
Source: Nat Commun. 2022 Nov 14;13:6913. doi: 10.1038/s41467-022-34457-5 (PMC9663431; doi:10.1038/s41467-022-34457-5)
Supplement: Supplementary file 1 — Reporting Summary [file 41467_2022_34457_MOESM1_ESM.pdf]

nature portfolio

Corresponding author(s): Andreas Nieder

Last updated by author(s): October 16, 2022

Reporting Summary

Nature Portfolio wishes to improve the reproducibility of the work that we publish. This form provides structure for consistency and transparency in reporting. For further information on Nature Portfolio policies, see our Editorial Policies and the Editorial Policy Checklist.

Statistics

For all statistical analyses, confirm that the following items are present in the figure legend, table legend, main text, or Methods section.

n/a	Confirmed	
☐	☒	The exact sample size (*n*) for each experimental group/condition, given as a discrete number and unit of measurement
☐	☒	A statement on whether measurements were taken from distinct samples or whether the same sample was measured repeatedly
☐	☒	The statistical test(s) used AND whether they are one- or two-sided<br>*Only common tests should be described solely by name; describe more complex techniques in the Methods section.*
☐	☒	A description of all covariates tested
☐	☒	A description of any assumptions or corrections, such as tests of normality and adjustment for multiple comparisons
☐	☒	A full description of the statistical parameters including central tendency (e.g. means) or other basic estimates (e.g. regression coefficient) AND variation (e.g. standard deviation) or associated estimates of uncertainty (e.g. confidence intervals)
☐	☒	For null hypothesis testing, the test statistic (e.g. F, t, r) with confidence intervals, effect sizes, degrees of freedom and P value noted<br>*Give P values as exact values whenever suitable.*
☒	☐	For Bayesian analysis, information on the choice of priors and Markov chain Monte Carlo settings
☐	☒	For hierarchical and complex designs, identification of the appropriate level for tests and full reporting of outcomes
☒	☐	Estimates of effect sizes (e.g. Cohen's *d*, Pearson's *r*), indicating how they were calculated

Our web collection on statistics for biologists contains articles on many of the points above.

Software and code

Policy information about availability of computer code

Data collection	CORTEX Version 595, Plexon MAP
Data analysis	MATLAB, MathWorks, R2020b

For manuscripts utilizing custom algorithms or software that are central to the research but not yet described in published literature, software must be made available to editors and reviewers. We strongly encourage code deposition in a community repository (e.g. GitHub). See the Nature Portfolio guidelines for submitting code & software for further information.

Data

Policy information about availability of data

All manuscripts must include a data availability statement. This statement should provide the following information, where applicable:

- Accession codes, unique identifiers, or web links for publicly available datasets
- A description of any restrictions on data availability
- For clinical datasets or third party data, please ensure that the statement adheres to our policy

The data that support the findings of this study are available from the corresponding author upon reasonable request. Source data are provided with this paper.

The datasets and code supporting the current study have not been deposited in a public repository because of further analyses, but are available by reasonable request to the corresponding author, Andreas Nieder (andreas.nieder@uni-tuebingen.de).

Human research participants

Policy information about studies involving human research participants and Sex and Gender in Research.

Reporting on sex and gender	n.a.
Population characteristics	n.a.
Recruitment	n.a.
Ethics oversight	n.a.

Note that full information on the approval of the study protocol must also be provided in the manuscript.

Field-specific reporting

Please select the one below that is the best fit for your research. If you are not sure, read the appropriate sections before making your selection.

☒ Life sciences ☐ Behavioural & social sciences ☐ Ecological, evolutionary & environmental sciences

For a reference copy of the document with all sections, see nature.com/documents/nr-reporting-summary-flat.pdf

Life sciences study design

All studies must disclose on these points even when the disclosure is negative.

Sample size	Two subjects, hundreds of neurons. Sample sizes were chosen not by statistical methods but rather to be comparable to typical sample sizes in the field for this type of research with highly trained animals.
Data exclusions	None
Replication	Data taken from randomly recorded neurons in two different subjects. All attempts at replication were successful.
Randomization	Stimuli and stimulus factors were presented in a pseudorandom order. Subjects were randomly selected.
Blinding	Owing to the exploratory nature of our study, we did not use randomization and blinding of animals.

Reporting for specific materials, systems and methods

We require information from authors about some types of materials, experimental systems and methods used in many studies. Here, indicate whether each material, system or method listed is relevant to your study. If you are not sure if a list item applies to your research, read the appropriate section before selecting a response.

Materials & experimental systems

n/a	Involved in the study	
☒	☐	Antibodies
☒	☐	Eukaryotic cell lines
☒	☐	Palaeontology and archaeology
☐	☒	Animals and other organisms
☒	☐	Clinical data
☒	☐	Dual use research of concern

Methods

n/a	Involved in the study	
☒	☐	ChIP-seq
☒	☐	Flow cytometry
☒	☐	MRI-based neuroimaging

Animals and other research organisms

Policy information about studies involving animals; ARRIVE guidelines recommended for reporting animal research, and Sex and Gender in Research

Laboratory animals	Carrion crows (Corvus corone), 2 males, 2 and 7 years
Wild animals	No wild animals were used in this study.

Reporting on sex	2 male crows
Field-collected samples	No field collected samples were used in this study.
Ethics oversight	All procedures were carried out according to the guidelines for animal experimentation and approved by the responsible national authorities, the Regierungspräsidium Tübingen, Germany.

Note that full information on the approval of the study protocol must also be provided in the manuscript.
